# Attempt of Real-Time Near-Infrared Fluorescence Imaging Using Indocyanine Green (ICG) in Radical Resection of Gallbladder Cancer: A Case Report

**DOI:** 10.3389/fsurg.2021.655805

**Published:** 2021-09-16

**Authors:** Yang Yu, Lin Xiang, Yuping Bai, Ewetse Paul Maswikiti, Baohong Gu, Xuemei Li, Haiyuan Li, Peng Zheng, Ying Zhang, Hao Chen

**Affiliations:** ^1^Department of Tumor Surgery, Lanzhou University Second Hospital, Lanzhou, China; ^2^The Second Clinical Medical College, Lanzhou University, Lanzhou, China; ^3^Department of Laboratory Medicine, the First Medical Centre, Chinese PLA General Hospital, Beijing, China

**Keywords:** near-infrared fluorescence, indocyanine green, gallbladder cancer, fluorescence laparoscope, case report

## Abstract

Surgery is the mainstay of treatment for resectable gallbladder cancer. Near-infrared fluorescence (NIRF) imaging using ICG is an innovation in laparoscopic surgery, which can provide real-time navigation during the whole operation. In this article, we present a 56-year older woman with gallbladder cancer, in which we evaluated the applicability of NIRF imaging using ICG for tumor and biliary tree visualization during the operative procedure of gallbladder cancer. The tumor and biliary tree were clearly visualized by utilizing a green fluorescence dye. The patient was successfully operated radical resection of gallbladder cancer under fluorescence laparoscope, without any complications. According to this case, the utilization of ICG based NIRF imaging is feasible and beneficial in identifying tumors and the biliary tree during radical resection. It can assist in the achievement of a negative margin and lymphatic clearance around the biliary tree. However, further studies are needed to corroborate the results of this case.

## Introduction

Gallbladder cancer (GBC) is a rare malignant tumor with a poor prognosis. GBC is the most common cancer arising from the biliary tract. The incidence of GBC is geographically variable, with high rates in South American countries, particularly Chile, Bolivia, and Ecuador, as well as some areas in India, Pakistan, Japan, and Korea (1, 2). In recent years, the incidence of gallbladder cancer showed an increasing tendency in the young generation. Surgery is the mainstay treatment for resectable GBC. Radical resection of GBC is a complex surgery whose procedure includes radical resection of the gallbladder, partial liver resection, and regional lymphadenectomy. The critical point of GBC surgery is to remove all lesions as much as possible while keeping an adequate remnant liver. However, it is challenging during surgery to estimate the extent of resection, the adequacy of surgical margin as well as the lymphatic clearance around the porta hepatis. GBC patients often have a poor prognosis due to the incomplete resection of lesions. Therefore, an intraoperative technique that can accurately identify the presence of microlesions is required for the guidance of operation. Near-infrared fluorescence (NIRF) imaging is an innovative technique with the ability of real-time tumor imaging during the operation. NIRF imaging can be utilized after intravenous injection of indocyanine green (ICG). ICG is a near-infrared fluorescent dye, which can be activated by foreign light with a wavelength of 750 ~810 nm and exhibit near-infrared light with an approximate wavelength of 840 nm (3). Almost all intravenous ICG is excreted through the biliary system. The presence of tumors and lesions causes local aggregation of ICG; then, this tumor-derived fluorescence can be detected by near-infrared technique during the surgical process. In recent years, due to its good optical properties and biological safety, NIRF imaging using ICG has been widely utilized as intraoperative assistance in the surgery of various malignant tumors (4–6). However, its application in gallbladder cancer surgery is still at a preliminary stage, with relatively few reports. In this case scenario, we describe the feasibility and effectiveness of NIRF imaging using ICG for tumor and biliary tree visualization during radical resection of gallbladder cancer. We present the following case in accordance with the CARE reporting checklist.

## Case Presentation

**Figure 1:**
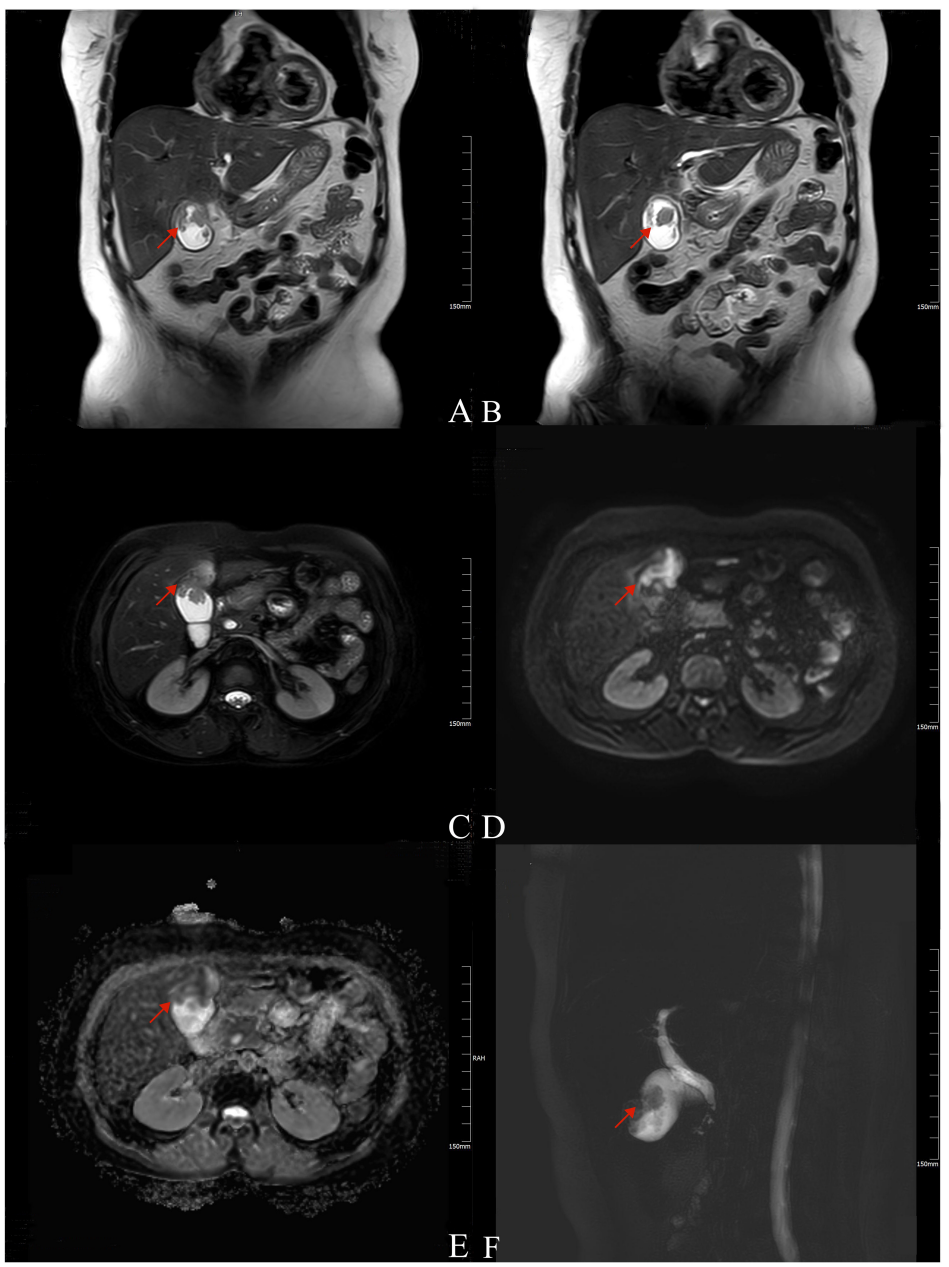
Magnetic resonance imaging (MRI) findings of the patient. T2-weighted **(A,B**, coronal; **C**, axial**)** imaging showed low signal intensity as an irregular filling defect in the gallbladder fundus and long signal in segment IV of the near liver (arrow). DWI **(D)** showed a hyperintense signal, and ADC map **(E)** showed reduced diffusion (arrow). MRCP **(F)** showed gallbladder enlargement with local filling defect (arrow).

A 56-year older woman was referred to our hospital presenting with a space-occupying lesion of the gallbladder. Prior to this, she was admitted to a local hospital complaining of pain localized in the right upper quadrant. Gallbladder cancer was confirmed according to an enhanced CT scanning imaging technique. For further workup and management, the patient was referred to our university hospital. In our hospital, she went through some systematic and detailed investigations. MRI revealed a gallbladder space-occupying lesion with an abnormal signal in segment IV of the liver, considered gallbladder cancer including liver invasion (Figure 1). Liver function tests were abnormal: TBIL 89.0 umol/L, DBIL 13.9 umol/L, IBIL 75.1 umol/L, ALP 69 U/L, γ-GGT 37 U/L, ALT 216 U/L, and AST 349 U/L; serum tumor marks tests showed an elevated CA-199. The results of these examinations supported the clinical diagnosis of gallbladder cancer. The patient was scheduled for surgical treatment.

Members of our multidisciplinary team convened to discuss the treatment and workup plan for this patient. The patient was diagnosed with gallbladder cancer (cT_3_N_X_M_0_). A consensus was reached that the radical surgery of gallbladder cancer is feasible and beneficial for this patient. Fluorescence laparoscopic surgery using ICG was proposed based on the assumption that it could help tumor visualization. The conventional preoperative preparations were performed. In addition, ICG (concentration 2.5 mg/ml) was administered intravenously to the patient 24 h prior to surgery. The dosage of ICG was calculated and administered as 0.1 mg per kg.

**Figure 2:**
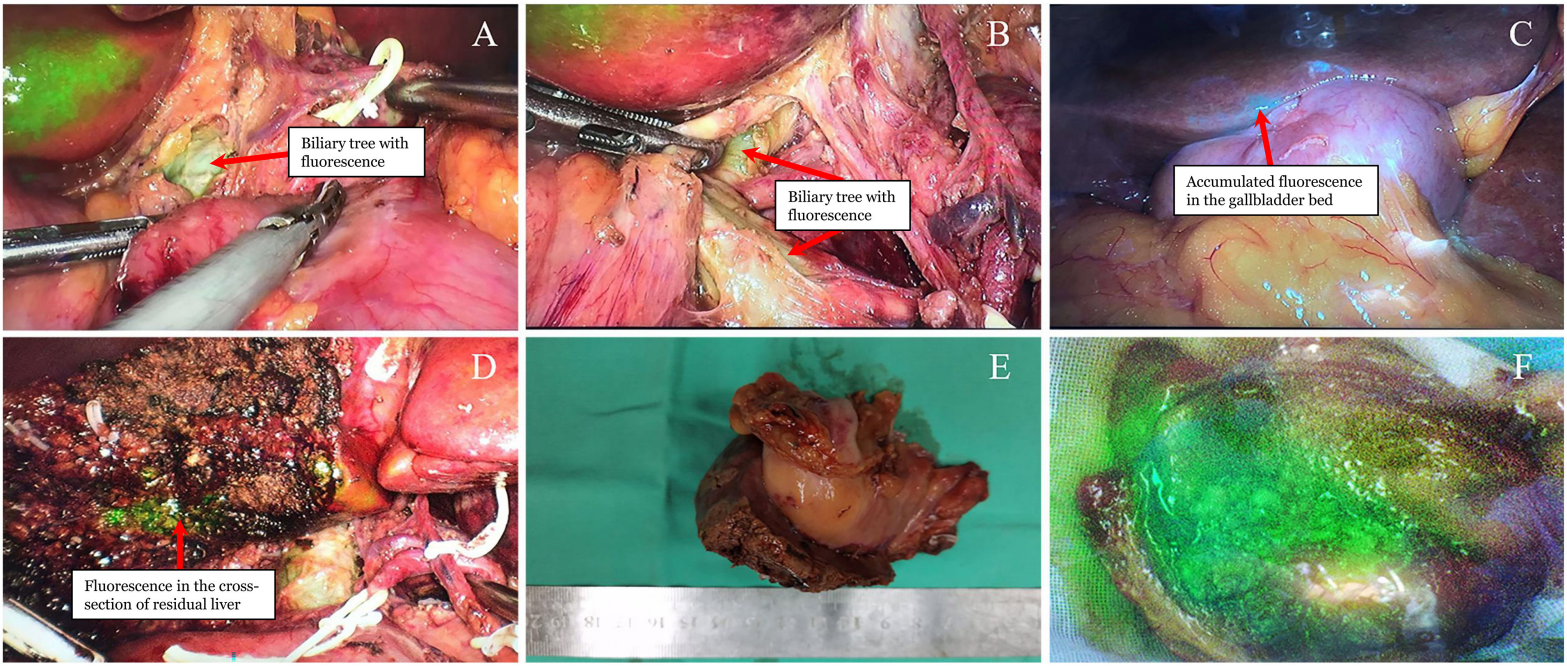
Visualization of the tumor and biliary tree under real-time NIRF imaging. **(A,B)** Pale green fluorescence detected in the extrahepatic biliary tree (arrow). **(C)** Green fluorescence accumulated into the gallbladder bed (arrow). **(D)** Residual green fluorescence observed after wedge liver resection (arrow). **(E)** Resected gallbladder and liver under the natural light. **(F)** Dense green fluorescence of the opened gallbladder under the NIFR imaging.

During the operation, the patient was placed in the scissors position. A small incision was made along the umbilical region to introduce a 12 mm trocar, and a pneumoperitoneum was then established with carbon dioxide gas. A laparoscope was inserted and introduced for an exploration surgical procedure to be carried out. Since there was no sign of distant metastasis, four additional trocars were placed under direct vision. Real-time NIRF imaging was achieved for the whole operative procedures via the PINPOINT Endoscopic Imaging System (Stryker). The extrahepatic biliary tree appeared pale green fluorescence under the NIRF imaging technique. Post identifying the extrahepatic biliary tree, the hepatic hilum was dissected. The hepatic arteries and portal veins were then isolated and protected (Figures 2A,B). At this stage, lymphadenectomy was performed, including the clearance of lymph nodes around the hepatoduodenal ligament (No.12), common hepatic artery (No.8), retropancreatic area (No.13), and celiac artery (No.9). Dissection was also done to view the Calot's triangle more vividly. The cystic artery was then ligated. To assure a negative cystic duct margin, the cystic duct was traced and transected to the common bile duct junction under the guidance of green fluorescence. Accumulation of fluorescence was identified along the gallbladder bed with the incorporation of NIRF imaging, which indicated a tumor invasion of the liver (Figure 2C). The wedge liver resection was performed 2-cm from the fluorescent accumulation margin. Subsequently, the wedge liver and gallbladder were staffed into the retrieval bag and then pulled out through the camera port. The cross-section of the reserved liver was explored and viewed under NIRF imaging, and local green fluorescence was observed (Figure 2D). Consequently, additional liver resection was added to clear the residual lesions. Finally, hemostasis was performed. The removed gallbladder specimen was dissected and observed under NIRF imaging once more. The tumor showed a dense green fluorescence under NIRF imaging (Figures 2E,F). There were no complications during the operative procedure, which lasted for 2 h, and 100 ml of blood was recorded.

**Figure 3:**
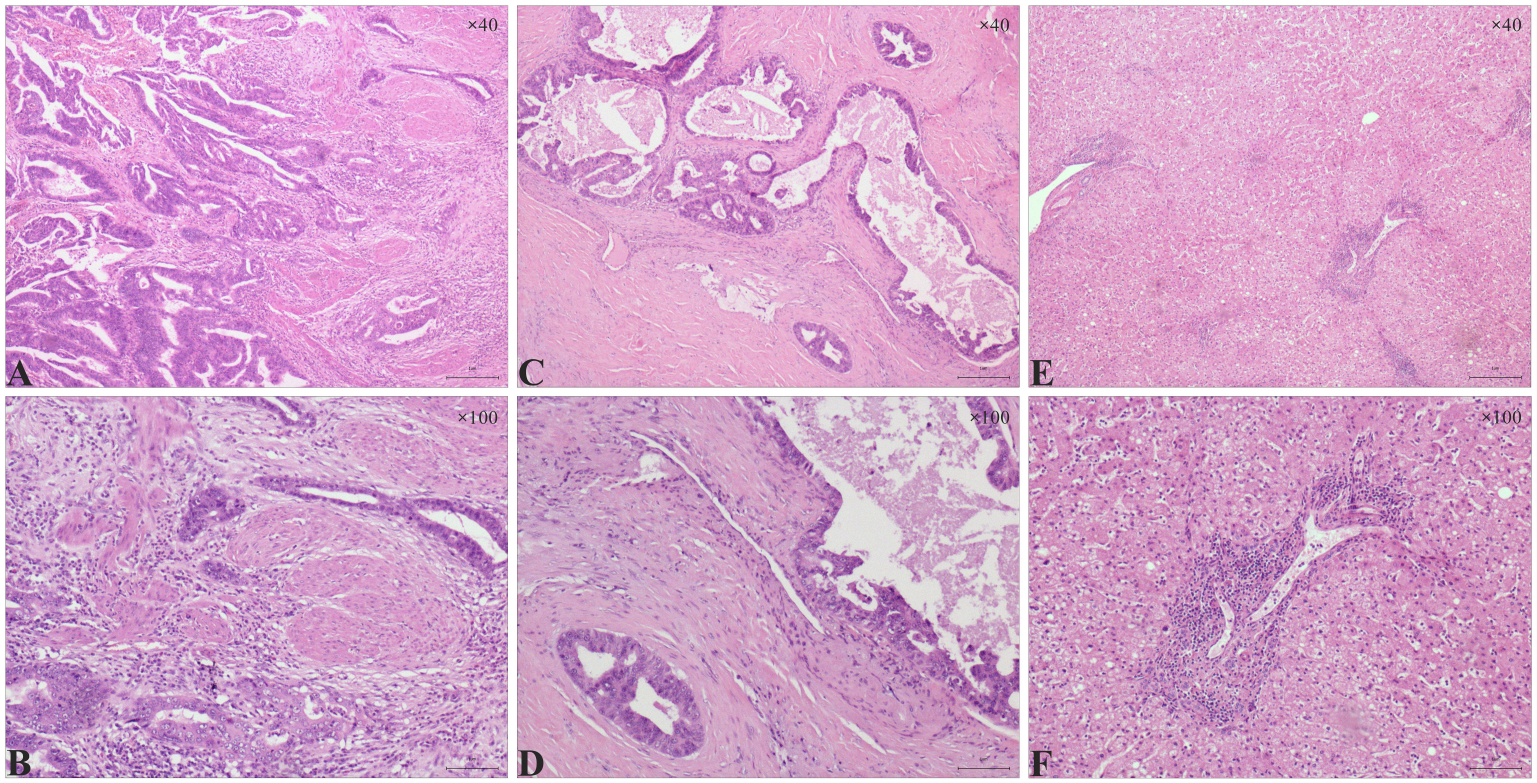
Histologic features in gallbladder adenocarcinoma and liver sections. **(A, B)** Stromal infiltration of gallbladder adenocarcinoma. **(A)** Atypical glands were observed in a low to moderately differentiated gallbladder adenocarcinoma **(A**, H&E, **×**40**)**. **(B)** Distorted glands invaded the muscularis propria layer of the gallbladder **(B**, H&E, ×100**)**. **(C,D)** Vascular invasion in gallbladder adenocarcinoma. **(C)** part of the dysplastic glands were sieve-like with mucus **(C**, H&E, ×40**)**. **(D)** Dysplastic glands invaded the vascular walls **(D**, H&E, ×100**)**. (**E,F)** A large number of lymphocytes infiltrated in the portal area of the liver; milder hepatic steatosis was observed in some areas **(E**, H&E, ×40; **F**, H&E, ×100**)**.

Finally, a pathological report revealed a 5 × 2.5 × 1 cm, low to moderately differentiated adenocarcinoma (Figure 3). The tumor invaded the whole layer of the gallbladder wall and has lymphovascular invasions. No tumor extension was identified in the liver. The resection margin of the cystic duct was free of tumors. No metastatic carcinoma in any lymph nodes (*n* = 6) was reported.

**Figure 4:**
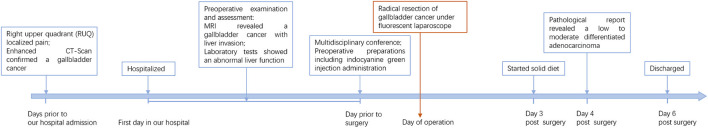
The timeline of patient information.

This patient recovered quickly post operatively. She started a solid diet on the 3rd day postoperatively based on her full recovery of bowel motions and activity. No postoperative complications occurred during her hospitalization stay. The patient was discharged on the 6th postoperative day and was asked to come back regularly for the following treatments. The detailed timeline of patient information in this episode of care can be seen in Figure 4.

## Discussion and Conclusions

GBC has a poor prognosis, with a 5-year survival rate approximating to 19% (7). Radical surgical resection is an integral component in the management of resectable GBC. The critical point of GBC surgery is to assure the complete removal of lesions and reduce the chances of recurrence. However, due to GBC's features of malignant growth, dense adhesions and obliteration of normal anatomic tissue planes are common in GBC patients, especially those complicated with inflammatory responses. This intricate operating environment requires a surgeon to be more skilled and precise during the operative procedure. In conventional views, open radical cholecystectomy is recommended for GBC surgery because it is more convenient to expose and operate during an open surgical approach. The laparoscopic approach is considered to have the possibility of increasing the risk of implantation metastasis in the trocar port and peritoneum; thus, the widespread use of laparoscope for GBC resection is challenging. However, the latest reports showed that laparoscopic radical cholecystectomy by an experienced surgeon had comparable oncologic safety and long-term outcomes than using open surgical approaches (8, 9). In addition, laparoscopic approaches have the advantages of less trauma and faster recoveries. Because of this, the laparoscopic approach is gradually being welcomed and accepted in radical resections of the GBC. In recent years, laparoscope-based assistance surgery systems are developing rapidly, such as the real-time NIRF imaging system, making the advantages of endoscopic techniques more obvious.

NIRF imaging for surgical treatment has been widely reported, especially its application to the liver and biliary tract surgeries (10, 11). The use of this technique involves a solution named ICG. ICG is a water-soluble compound, which exhibits fluorescence in the near-infrared light spectrum of approximately 800 nm wavelength. After intravenous administration, ICG is ingested by hepatocytes and expelled out almost entirely through the biliary system within a few hours. It was widely reported that ICG would be retained at the lesion site of liver tumor and can exhibit continuous green fluorescence under the near-infrared light detector[10]. The underlying mechanism may be that after tumor occurrence, the portal vein remains a normal function of ICG uptake, but the biliary tract has impaired functions in the excretion of ICG. In addition, tumor including GBC, can be specifically imaged by ICG due to the tissue-non-specific delivery of the dye and preferential tumor cellular uptake (12, 13). ICG has good biological safety and is the only near-infrared imaging drug approved by the US Food and Drug Administration (FDA). Based on these advantages, ICG is utilized together with the near-infrared laparoscopy, making real-time visualization of tumors become a reality during the hepatobiliary tumor resection.

At present, the utilization of NIRF imaging with ICG has been rarely reported in the radical surgery of GBC (14). Based on our center's experience in the field of fluorescent laparoscopy, the utilization of fluorescent laparoscopy in radical resection of GBC was preliminarily tested in this case. ICG was administered to the patient 1 day before surgery as the previous reports had confirmed a good safety and efficiency of the preoperative protocol with intravenous administration in tumor visualization and intraoperative cholangiography for hepatobiliary surgery (15, 16). In our case, both the tumor and extrahepatic biliary tree were successfully and clearly imaged during the operation. The real-time imaging of the tumor and extrahepatic biliary tree provided navigation for the operation of key steps. ICG is excreted through the biliary tree, which showed green fluorescence under NIRF imaging. The fluorescence of the extrahepatic biliary tree came from the flowing ICGs that were getting excreted, which is different from the fluorescence from the accumulated ICGs in the tumor. It appeared pale green due to the low ICG concentration. Due to the achievement of biliary tree imaging, some critical structures such as the bile ducts and the surrounding non-fluorescent hepatic arteries, along with any aberrant anatomy, can be quickly identified. As a result, the dissection of the hepatic hilum became more accurate and efficient. All of these allowed for the accurate identification and complete removal of the hilar lymph nodes. Finally, a total of 6 lymph nodes were well removed in this case. However, limitations of our fluorescence-guided protocol have to been pointed out in some special conditions. Although the extrahepatic biliary tree was successfully imaged with the pale fluorescence in our case, this pale fluorescence can be challenging in helping identification of the bile duct when there are severe inflammatory adhesions or anatomical distortion, especially in case of reoperation for postoperatively diagnosed GBC. This suggested that the timing of ICG administration should be optimized or a better protocol was needed to enhance the imaging of extrahepatic biliary tree. For example, the protocol of twice administrations can be utilized that an additional ICG administration is performed close to the beginning of operation due to most of ICGs excrete through the bile duct 30–60 min after intravenous injection.

On the other hand, intraoperative imaging of the tumor also provides critical guidance for operative procedures. First, tumor imaging ensured that the tumor-free operation principle has well adhered during resection of the tumor. With the aid of NIRF imaging, tumors with real-time green fluorescence can be easily identified, and thus unnecessary intraoperative tumor contact can be avoided. Second, the accumulation of green fluorescence in the gallbladder bed helped determine the extent of liver resection. To date, wedge liver resection is generally recommended for early liver invasion. However, there is some controversy over the extent of wedge resection. In different institutions, the liver resection extent varied from 1 to 5cm distance between tumor and resection margin (17–22). For the determination of wedge liver extent, the most important parameter is to identify the tumor boundary. In many cases, identifying the tumor boundary is not easy in normal vision because liver metastasis is sometimes small or located inside the liver. For instance, during the first reported laparoscopic radical cholecystectomy of GBC, the medical transection line was determined based on the surgeon's subjective judgment when performing a wedge resection of the gallbladder bed; however, the medial margin along segment IVB was found to be positive on frozen section analysis, which ended up leading the surgeon to perform a second transection operative procedure (23). Although NIR fluorescence along the gallbladder bed can also result from local bile stasis caused by the surrounding gallbladder tumor, we still suspected our case more of the possibility of liver invasion due to positive findings of the same location in preoperative MRI. Thus, the boundary of liver invasion was determined based on the fluorescence imaging technology, and wedge liver resection with a 2 cm margin from the boundary was performed. After liver wedge resection, local fluorescence accumulation was still observed in the section of the preserved liver, indicating possible tumor residue. Subsequently, an additional lesion resection was added to assure a completely negative margin. However, the postoperative pathological report revealed some unexpected results. There were no tumor extensions identified in the liver. After discussing with the pathologist, we reached a consensus that the patient had a strong possibility of liver tumor extension although it is not identified from the pathological sections likely due to small lesions. Therefore, a reasonable explanation can be given that local micro invasion of cancer cells damaged the local duct for the excretion of ICG, causing the liver surface-accumulated fluorescence. If this is the truth, it suggested that the fluorescence imaging technique has a good sensitivity to detect GBC, even to microlesions. However, we also can't neglect the false-positive possibility of NIRF imaging in detecting tumor cells. The fluorescence accumulation in the liver may come from local bile stasis that had no direct relationship with tumor extension. In some reports regarding the detection of metastatic or primary tumor with intraoperative NIRF imaging, sensitivity varied from 72.4 to 100%, false positive rate from 0 to 45.8% (24, 25). The occurrence of false positives suggested that it was hard to distinguish the fluorescence coming from real cancer or other conditions. Interventions for fluorescence imaging-identified tumors should be performed with caution, especially when false alarms can cause severe consequences.

The operation of this patient was smoothly performed, and it lasted for 2 h which is shorter than the operative time of conventional laparoscopic or open radical gallbladder surgery (26–29). The estimated blood volume was comparable to similar reports (14). The previous report showed that the rates of iatrogenic bile duct injuries during laparoscopic cholecystectomy is about 0.2–0.4% (30, 31). In the reported case, no intraoperative and postoperative complications were observed during her treatment course. In addition, the advantages of minimally invasive operation, including less trauma and rapid recovery, made the patient have a good performance status to receive subsequent therapy smoothly. This case showed great success with support from the intraoperative visualization by NIRF imaging.

In conclusion, this case provides a preliminary attempt of employing ICG based NIRF imaging for tumor and biliary tree visualization in gallbladder cancer surgery. Our experience from this case indicated that using ICG-based NIRF imaging appeared to be advantageous and safe in complex minimally invasive surgeries. It can assist in the achievement of negative margin and lymphatic clearance around the biliary tree during the radical resection process of gallbladder cancer surgery. However, it should be careful when adapting these results to other patients due to the limitations of single case studies. More studies on large samples are needed to further corroborate our results.

## Data Availability Statement

The original contributions presented in the study are included in the article/supplementary material, further inquiries can be directed to the corresponding authors.

## Ethics Statement

Ethical review and approval was not required for the study on human participants in accordance with the local legislation and institutional requirements. The patients/participants provided their written informed consent to participate in this study. Written informed consent was obtained from the individual(s) for the publication of any potentially identifiable images or data included in this article.

## Author Contributions

HC and YZ had the idea of this report. YY collected the patient's clinical data, analyzed the data, and drafted the manuscript. HL, PZ, and HC performed the surgical procedure. BG, XL, EM, and YZ performed the literature search and data summarization. LX conducted the histopathologic examination and acquired the photos. YB collected and analyzed the MR imaging. All authors contributed to the revision of the manuscript and approved the submitted manuscript.

## Funding

This work was supported by the Key Talents Project of Gansu Province (2019RCXM020); Key Project of Science and Technology in Gansu province (19ZD2WA001); Science and Technology Project of Chengguan District of Lanzhou City (2019RCCX0034); and Cuiying Scientific and Technological Innovation Program of Lanzhou University Second Hospital (CY2017-ZD01).

## Conflict of Interest

The authors declare that the research was conducted in the absence of any commercial or financial relationships that could be construed as a potential conflict of interest.

## Publisher's Note

All claims expressed in this article are solely those of the authors and do not necessarily represent those of their affiliated organizations, or those of the publisher, the editors and the reviewers. Any product that may be evaluated in this article, or claim that may be made by its manufacturer, is not guaranteed or endorsed by the publisher.

## Abbreviations

ICG: indocyanine green
NIRF: Near-infrared fluorescence
GBC: Gallbladder cancer
MRI: Magnetic resonance imaging
FDA: the US Food and Drug Administration.
